# A special issue on new insights into genome maintenance

**DOI:** 10.1186/s13578-017-0137-7

**Published:** 2017-01-31

**Authors:** Guo-Min Li

**Affiliations:** Department of Biochemistry and Molecular Biology, Norris Comprehensive Cancer Center, University of Southern California Keck School of Medicine, Los Angeles, CA USA

Maintaining genome stability is essential for preventing various human diseases including cancer. Previous studies have elucidated multiple cellular mechanisms for genome maintenance, which can be classified into two major groups: one that deals with replication-associated abnormalities, and the other that repairs various DNA lesions. The replication fidelity maintenance mechanisms involve DNA polymerases and the DNA mismatch repair pathway. While replicative DNA polymerases and the mismatch repair system are responsible for correcting mispairs generated during DNA replication [1, 2], translesion DNA polymerases ensure uninterrupted DNA replication by bypassing template strand DNA lesions [3], which can be removed after the completion of DNA synthesis. The DNA repair pathways, which include base excision repair [4], nucleotide excision repair [5, 6], double strand break repair [7–9], and inter-strand crosslink repair [10, 11], remove essentially all kinds of DNA lesions. These discoveries have led to the current understanding of cellular response to DNA damage, and have earned the field many remarkable awards, including the 2015 Nobel Chemistry Prize to Tomas Lindahl, Paul Modrich and Aziz Sancar [12–15], and the 2015 Lasker Basic Medical Research Award to Stephen Elledge and Evelyn Witkin [16].

Building on the previous discoveries, recent investigations in the field have revealed new insights into the mechanisms of the genome maintenance systems. In this thematic series, *Cell and Bioscience* presents a series of reviews attempting to provide an overview of the latest breakthroughs and developments in the field. Specifically, this series focuses on (1) novel regulation of DNA damage response by ubiquitinating and deubiquitinating enzymes (He et al.); (2) the impact of bulky DNA lesions on error-prone or error-free transcription (Shin et al.); (3) the genome maintenance function of Fanconi anemia proteins (Palovcak et al.); (4) new factors and mechanisms of DNA break end joining (Wang and Xu); (5) mutagenic and tumorigenic activities of APOBEC3B (Peng et al.); and (6) nonsense RNA-mediated cellular surveillance pathway (Nickless et al.).

It is our sincere hope that this thematic series brings our readers enlightenment and offers sufficient introductory information to help them appreciate the new breakthroughs and developments in the field.

## References

[1] Kolodner RD (2016). A personal historical view of DNA mismatch repair with an emphasis on eukaryotic DNA mismatch repair. DNA Repair.

[2] Kunkel TA, Erie DA (2015). Eukaryotic mismatch repair in relation to DNA replication. Ann Rev Genet.

[3] Lange SS, Takata K, Wood RD (2011). DNA polymerases and cancer. Nat Rev Cancer.

[4] Hegde ML, Hazra TK, Mitra S (2008). Early steps in the DNA base excision/single-strand interruption repair pathway in mammalian cells. Cell Res.

[5] Fousteri M, Mullenders LH (2008). Transcription-coupled nucleotide excision repair in mammalian cells: molecular mechanisms and biological effects. Cell Res.

[6] Shuck SC, Short EA, Turchi JJ (2008). Eukaryotic nucleotide excision repair: from understanding mechanisms to influencing biology. Cell Res.

[7] Pannunzio NR, Li S, Watanabe G, Lieber MR (2014). Non-homologous end joining often uses microhomology: implications for alternative end joining. DNA Repair.

[8] Weterings E, Chen DJ (2008). The endless tale of non-homologous end-joining. Cell Res.

[9] Ceccaldi R, Rondinelli B, D’Andrea AD (2016). Repair pathway choices and consequences at the double-strand break. Trends Cell Biol.

[10] Shen X, Li L (2010). Mutagenic repair of DNA interstrand crosslinks. Environ Mol Mutagen.

[11] Deans AJ, West SC (2011). DNA interstrand crosslink repair and cancer. Nat Rev Cancer.

[12] Li GM (2016). Celebrating the work of Nobel Laureate Paul Modrich. Sci China Life Sci.

[13] Mi S, Klungland A, Yang YG (2016). Base-excision repair and beyond—a short summary attributed to scientific achievements of Tomas Lindahl, Nobel Prize Laureate in Chemistry 2015. Sci China Life Sci.

[14] Orren DK (2016). The nobel prize in chemistry 2015: exciting discoveries in DNA repair by Aziz Sancar. Sci China Life Sci.

[15] Li GM (2016). A personal tribute to 2015 nobel laureate Paul Modrich. DNA Repair.

[16] Zou L, Li L (2016). The 2015 Albert Lasker basic medical research award: an exhilarating journey to the DNA damage checkpoint. Sci China Life Sci.

